# Reduced sleep time is associated with increases in frontal sleep-like activity and emotion regulation failures

**DOI:** 10.1192/j.eurpsy.2021.452

**Published:** 2021-08-13

**Authors:** G. Avvenuti, D. Bertelloni, G. Lettieri, E. Ricciardi, L. Cecchetti, P. Pietrini, G. Bernardi

**Affiliations:** 1 Momilab - Space Group, IMT School for Advanced Studies, Lucca, Italy; 2 University Of Pisa, University of Pisa, Pisa, Italy; 3 Momilab - Sane Group, IMT School for Advanced Studies Lucca, Lucca, Italy; 4 Momilab, IMT School for Advanced Studies, Lucca, Italy

**Keywords:** EEG, emotion regulation, behavior, sleep

## Abstract

**Introduction:**

Emotion self-regulation relies both on cognitive and behavioral strategies implemented to modulate the subjective experience and/or the behavioral expression of a given emotion.

**Objectives:**

While it is known that a network encompassing fronto-cingulate and parietal brain areas is engaged during successful emotion regulation, the functional mechanisms underlying failures in emotion suppression are still unclear.

**Methods:**

We analyzed facial-view video and high-density EEG recordings of nineteen healthy adult subjects (26±3yrs, 10F) during an emotion suppression (ES) and a free expression (FE) task performed on two consecutive days. An actigraph was worn for 7-days and used to determine sleep-time before each experiment. Changes in facial expression were identified and manually marked on the video recordings. Continuous hd-EEG recordings were preprocessed using standard approaches to reduce artifactual activity and source-modeled using sLORETA.

**Results:**

Changes in facial expression during ES, but not FE, were preceded by local increases in sleep-like activity (1-4Hz) in in brain areas responsible for emotional suppression, including bilateral anterior insula and anterior cingulate cortex, and in right middle/inferior frontal gyrus (p<0.05, corrected; Figures 1 and 2). Moreover, shorter sleep duration the night prior to the ES experiment correlated with the number of behavioral errors (p=0.01; Figure 3) and tended to be associated with higher frontal sleep-like activity during emotion suppression failures (p=0.05).

**Conclusions:**

These results indicate that local sleep-like activity may represent the cause of emotion suppression failures in humans, and may offer a functional explanation for previous observations linking lack of sleep, changes in frontal activity and emotional dysregulation.

**Disclosure:**

No significant relationships.

